# Antiphospholipid Antibodies to Domain I of Beta-2-Glycoprotein I Show Different Subclass Predominance in Comparison to Antibodies to Whole Beta-2-glycoprotein I

**DOI:** 10.3389/fimmu.2018.02244

**Published:** 2018-09-28

**Authors:** Thomas McDonnell, Bahar Artim-Esen, Chris Wincup, Vera M. Ripoll, David Isenberg, Ian P. Giles, Anisur Rahman, Charis Pericleous

**Affiliations:** ^1^Division of Medicine, Rayne Institute, University College London, London, United Kingdom; ^2^Division of Rheumatology, Department of Internal Medicine, Istanbul Faculty of Medicine, Istanbul University, Istanbul, Turkey; ^3^Imperial College Vascular Sciences National Heart & Lung Institute, Imperial Centre for Translational and Experimental Medicine (ICTEM), London, United Kingdom

**Keywords:** antiphosholipid antibodies, IgG 3, Antiphospholid syndrome, domain I, Beta 2 glycoprotein

## Abstract

Antiphospholipid antibodies (aPL), the serological hallmark of antiphospholipid syndrome (APS), are a heterogeneous group of autoantibodies raised against circulating blood proteins. Of these proteins, the phospholipid-binding b2-glycoprotein I (β2GPI) is considered to be the main autoantigen in APS. Indeed, IgG antibodies targeting b2GPI (ab2GPI) directly cause both thrombosis and pregnancy morbidity in several mouse models. While antibodies raised against all five domains of b2GPI have been reported, a subgroup of IgG ab2GPI raised against the first domain (DI) of b2GPI (aDI), strongly correlate with thrombotic APS, and drive thrombosis and pregnancy loss *in vivo*. Few studies have focused on determining the type of IgG subclass(es) for aPL. The subclass of an antibody is important as this dictates the potential activity of an antibody; for example, IgG1 and IgG3 can fix complement better and are able to cross the placenta compared to IgG2 and IgG4. It is unknown what subclass IgG aDI are, and whether they are the same as ab2GPI. To determine IgG subclass distribution for ab2GPI and aDI, we purified total IgG from the serum of 19 APS patients with known ab2GPI and aDI activity. Using subclass-specific conjugated antibodies, we modified our established in-house ab2GPI and aDI ELISAs to individually measure IgG1, IgG2, IgG3, and IgG4. We found that while IgG1, IgG2, and IgG3 ab2GPI levels were similar, a marked difference was seen in IgG subclass aDI levels. Specifically, significantly higher levels of IgG3 aDI were detected compared to IgG1, IgG2, or IgG4 (*p* < 0.05 for all comparisons). Correlation analysis of subclass-specific ab2GPI vs. aDI demonstrated that IgG3 showed the weakest correlation (*r* = 0.45, *p* = 0.0023) compared to IgG1 (*r* = 0.61, *p* = 0.0001) and IgG2 (*r* = 0.81, *p* = 0.0001). Importantly, total subclass levels in IgG purified from APS and healthy serum (*n* = 10 HC *n* = 12 APS) did not differ, suggesting that the increased IgG3 aDI signal seen in APS-derived IgG is antigen-specific. To conclude, our data suggests that aDI show a different IgG subclass distribution to ab2GPI. Our results highlight the importance of aDI testing for patient stratification and may point toward differential underlying aPL-driven pathogenic processes that may be subclass restricted.

## Introduction

Antiphospholipid Syndrome is an autoimmune rheumatic disorder with an estimated population prevalence of between 0.3 and 1% of the population (1), with a female predominance. Often presenting in association with systemic lupus erythematosus (SLE, secondary APS), APS is the commonest cause of acquired hypercoagulability, accounting for one in six strokes in patients under 50 years old (2), one in nine heart attacks (3), and associated with an almost 3-fold greater risk of atherosclerosis, even in the absence of SLE (primary APS), compared to matched controls (4).

Antibodies play a key role in the pathogenesis of APS. The accepted international classification criteria for the disease (5) require positive tests for antiphospholipid antibodies (aPL) on at least two occasions, at least 12 weeks apart. There are three different tests routinely used to detect aPL in clinical practice. Two of these are enzyme-linked immunosorbent assays (ELISAs) for IgG and IgM antibodies to cardiolipin (aCL ELISA) or β2 glycoprotein I (aβ2GPI ELISA), while the third is a functional test that detects the effect of aPL on clotting time in the presence or absence of excess phospholipid (the lupus anticoagulant or LA test). aCL and aβ2GPI antibodies are found in various isotypes. Though only IgG and IgM measurements are defined in the classification criteria (5), there is increasing interest in the role played by IgA aPL. Despite this, the IgG isotype best correlates with clinical events in patients (6–8) and is the most often studied *in vitro* and *in vivo* (9–14).

There is extensive evidence from clinical studies and mouse models of both thrombosis and pregnancy loss (15–19) that IgG aPL play a direct pathogenic role in APS. However, not all aPL are equally pathogenic. McNeil et al (20) showed that non-pathogenic aPL found in healthy people could bind both neutral and anionic PL without the need for a co-factor, whereas aPL from patients with APS showed preferential binding to anionic PL but required the presence of the serum co-factor β2GPI. Subsequently, it was shown that these pathogenic aPL could bind β2GPI in the absence of PL. β2GPI has five domains, and multiple studies have shown that antibodies to the N-terminal domain (Domain I or DI, anti-DI antibodies or aDI) are most closely linked to development of thrombosis (12, 13, 21–24). Our group has shown that when IgG from patients with APS is fractionated into aDI-rich and aDI-poor fractions by affinity purification on a DI column and the fractions used to stimulate thrombosis in a mouse model of APS, the thrombogenic potential is concentrated in the aDI fraction (25). In the same mouse model, we also showed that recombinant DI can block induction of thrombosis by IgG from patients with APS IgG antibodies are produced in four subclasses: IgG1, IgG2, IgG3, and IgG4 (26). Each of these subclasses has different avidities, affinities and abilities, for example IgG1 and IgG3 can cross the placenta whilst IgG2 and IgG4 cannot. They also have different structures, with IgG3 displaying a far longer hinge region than the other subclasses. Their ability to interact with Fc receptors and their affinity for those receptors also differs, whilst their alternative structures lend themselves to different kinetics in serum, with IgG3 displaying a range of half-lives (27). In SLE, there is literature showing that IgG antibodies to key autoantigens are concentrated in certain subclasses, with confounding results in some cases. For example, Zahir et al. (28) studied 120 SLE patients and showed that IgG3 anti-nucleosome antibodies were present at high levels in active but not inactive SLE, rose during flares of disease activity and showed a particularly close association with nephritis. In contrast, Ravirajan et al. (29) showed that in 31 patients with SLE, anti-nucleosome were predominantly IgG2, anti-dsDNA antibodies were IgG1 and IgG3, and anti-heparan sulfate were IgG2 and IgG3. There is also a precedent for investigating IgG subclass distribution in APS with studies examining the subclass distribution of IgG aCL (30, 31), which seemingly appear to be of all four subclasses but with a potentially pathogenic predominance for IgG2 and IgG4. Previous research had suggested that IgG1 or IgG2 predominate for aβ2GPI (32, 33), however, no-one has investigated the subclass distribution of patient antibodies for IgG aDI subclasses.

This led us to test whether the more pathogenic aDI subgroup of aβ2GPI has a different subclass distribution compared to aβ2GPI as a whole.

## Methodology

### Patients and controls

Serum was obtained from 19 patients with APS and 5 healthy controls (HC). Serum samples were collected by informed consent following local institutional ethical approval from 19 patients from 2 centers: University College London [London, UK], University of Istanbul [Istanbul, Turkey]. All 19 patients fulfilled the revised classification criteria for APS (5).

### IgG purification

Polyclonal IgG was purified from serum of patients and healthy controls as follows. Serum was diluted with physiological phosphate buffer (pH 7.4) and run through a protein G sepharose column (Pierce) before elution with 0.1 M Glycine (pH 2.7) and neutralization with 1 M Tris buffer. Eluted IgG was desalted and dialysed in physiological phosphate buffer (pH 7.4) using a centrifugal concentrator. Samples were quantified by BCA (Pierce).

### Establishing an ELISA method for direct comparison of IgG subclass levels using optical density (OD) units

Determination of the appropriate conditions for detecting all four IgG subclasses was carried out using our in-house whole IgG ELISA. Briefly, anti- human IgG (Sigma, I8885) is coated at 400 ng/ml on half a maxisorp plate, the other half coated with PBS alone. Samples are diluted in PBS, plates are washed 3 times before application of sample with PBST (0.1%). Secondary antibody (anti-human IgG, HRP Conjugated) was applied at 1:1000 and incubated for 1 h at room temperature. Substrate was warmed to room temperature and applied to the plate post washing (100 μl) for 30 min. Plates were stopped with acid and read at 450 nM. Specificity for subclass was achieved using subclass specific HRP conjugated secondary antibodies (Sigma). Secondary antibody concentrations were determined using a serum standard of known subclass composition. All secondary antibodies were diluted to give OD values matching the expected outcome from the known serum.

Goat anti-human IgG against the Fc portion (I8885, Sigma) was used to coat maxisorp plates (400 ng/ml) overnight at 4°C. Plates were blocked with 2% BSA/PBS for 1 h at 37°C, and the commercial serum sample loaded at a dilution of 1:100000 in 1% BSA/PBS for 1 h at room temperature (RT). Detection was carried out using specific mouse anti-human secondary antibodies at a range of values from 1:1000 to 1:80000 for 1 h at RT. Optimal concentrations were as follows: anti-IgG1 at 1:1000, anti-IgG2 at 1:2000, anti-IgG3 at 1:10000 and anti-IgG4 at 1:80000. Plates were washed and substrate added and developed in the dark for 10 min. The reaction was stopped with 1% HCl and plates were read at 450nm.

### Measurement of total IgG1, IgG2, IgG3, and IgG4 in purified IgG samples

In order to be sure that differences between samples in the amount of each IgG subclass binding to β2GPI or DI were indeed antigen-specific findings and due to biasing of an individual subclass, we carried out an ELISA to measure total IgG1, IgG2, IgG3, or IgG4. In essence, this assay was the reverse of our whole IgG ELISA, as described above. Four separate lanes of a maxisorp plate were coated with mouse anti-human IgG1, IgG2, IgG3, or IgG4 respectively. After blocking, purified IgG samples from patients and controls were diluted to 500 μg/ml in 1% BSA/PBS and added to the plates, followed by incubation for 1 h at 37°C. Anti-human IgG secondary antibody conjugated to HRP (A6029, Sigma) was added for 1 h at 37 °C. Substrate was added for 10 min, the reaction was stopped with 1% HCL and OD was read at 450nm. We compared OD values obtained from 10 HC and 12 APS patients.

### aβ2GPI subclasses ELISA

To measure IgG subclass specific aβ2GPI, we employed our in-house aβ2GPI ELISA (35). Plates were prepared by coating overnight with 4 μg/ml of commercial purified human β2GPI at 4°C (Enzyme Research Laboratories). Plates were then blocked for 1 h at 37°C with 2% BSA/PBS. Purified IgG samples from patients and controls were prepared in 1% BSA/PBS at a protein concentration of 500 μg/ml. Samples were applied to the plate for 1 h before washing with PBS Tween (0.01%) and subclass specific secondary antibodies (as established above) applied. Secondary antibodies were diluted to the levels determined against the calibrant material. Incubation with secondary antibody was for 1 h before washing and application of substrate for 15 min, followed by addition of 1% HCl to stop the reaction. Samples were read at 450 nm. Binding for the individual subclasses was compared. For each patient, all four secondary antibodies against the four IgG subclasses were tested on the same plate.

### aDI subclasses ELISA

Similarly to the aβ2GPI assay, we utilized our in-house aDI ELISA to measure IgG subclass specific aDI (34). The assay was performed in exactly the same manner as per our aβ2GPI ELISA, with the exception of the DI plates being coated with 10 μg/ml of folded, conformationally correct DI for 2 h at 37°C. For each patient, all four secondary antibodies against the four IgG subclasses were tested on the same plate.

### Statistical analysis

Statistical analysis was carried out using Prism V5.0. We performed a 1 way ANOVA with a Kruskal-Wallis post test to determine significant differences between IgG subclass distributions. Linear regression analysis was carried out for correlating subclasses between the two antigens, β2GPI and DI.

## Results

Samples from 19 different patients with APS were tested. Their clinical and serological characteristics are shown in Table 1. Twelve patients with APS had a history of vascular thrombosis (VT) only, four had experienced pregnancy morbidity (PM) only, one had VT and PM and two had catastrophic antiphospholipid syndrome (CAPS).

**Table 1:** This table shows the characteristics of the patients and healthy controls included in the study.

	**APS**	**HC**
Age	36.8 (11.8)	33.3 (8.5)
Sex	12F, 6M	3F 1M
aβ2GPI	77.7 (30.4)	–
aDI	45.19 (35.9)	–
Other ARD	11	–
LA	13	–

*Mean age, aβ2GPI and aDI levels are shown, with standard deviations in parentheses. aβ2GPI positivity cut off: 8 GBU, aDI positivity cut off: 10 GDIU*.

### The pattern of IgG subclass detection is different for aβ2GPI compared to aDI

All 19 APS samples tested positive for aβ2GPI, 18 of which were positive for aDI. The mean activity for all samples is shown in Table 1.

**Figure 1:**
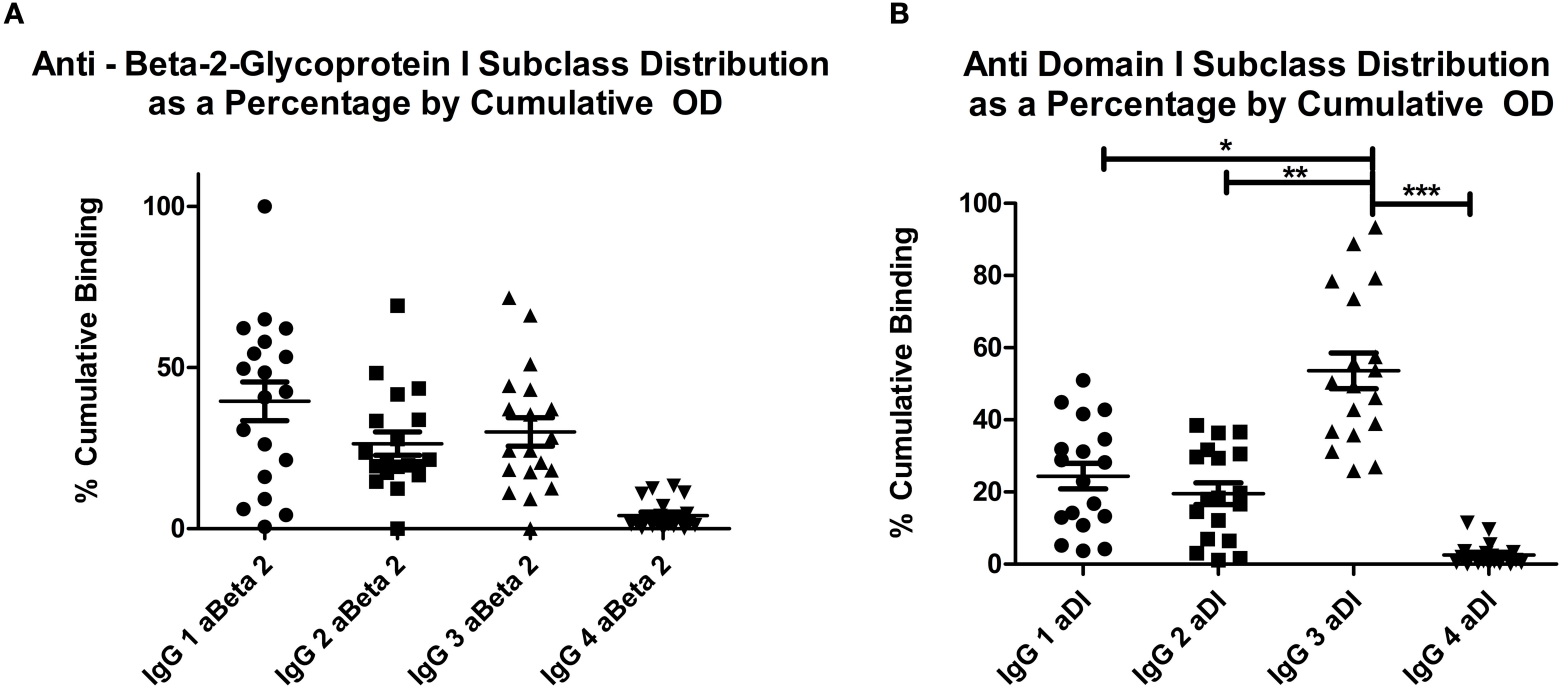
**(A)** shows the distribution of OD as a percentage of cumulative OD for aβ2GPI antibodies, **(B)** shows the same measure for aDI antibodies. The increase in IgG3 percentage can be seen in **(B)**, this was significantly higher than any other subclass. Both panels show IgG4 significantly lower than any other subclass for both antigens. *p < 0.05; ** *p* < 0.01; *** *p* < 0.001. IgG4 vs. IgG1, IgG2 or IgG3 in both **(A)** and **(B)** = *p* < 0.05.

Figures 1A,B show the relative percentages of subclass for each antigen (1A, aβ2GPI and 1B, aDI). This was calculated by expressing the OD for each subclass as a percentage of the cumulative OD generated from all four subclasses, for each individual patient. The mean OD, as well as the average percentage of cumulative binding for all four IgG subclasses in both aβ2GPI and aDI assays can be seen in Table 2.

**Table 2:** This table contains the raw OD and the average percentage of the cumulative OD for each subclass against both antigens.

	**a**β**2GPI**		**aDI**	
	**Mean OD (±*SD*)**	**Average % of cumulative binding (±*SD*)**	**Mean OD (±*SD*)**	**Average % of cumulative binding (±*SD*)**
IgG 1	0.6 (±0.7)	39.5 (±26.0)	0.3 (±0.6)	24.3 (±14.9)
IgG 2	0.31(±0.27)	26.4 (±15.7)	0.2 (±0.3)	19.5 (±12.7)
IgG 3	0.37 (±0.35)	29.9 (±19.1)	0.45 (±0.4)	53.5 (±21.0)
IgG 4	0.07 (±0.15)	4.1 (±4.5)	0.02 (±0.04)	2.5 (±3.3)

**Figure 2:**
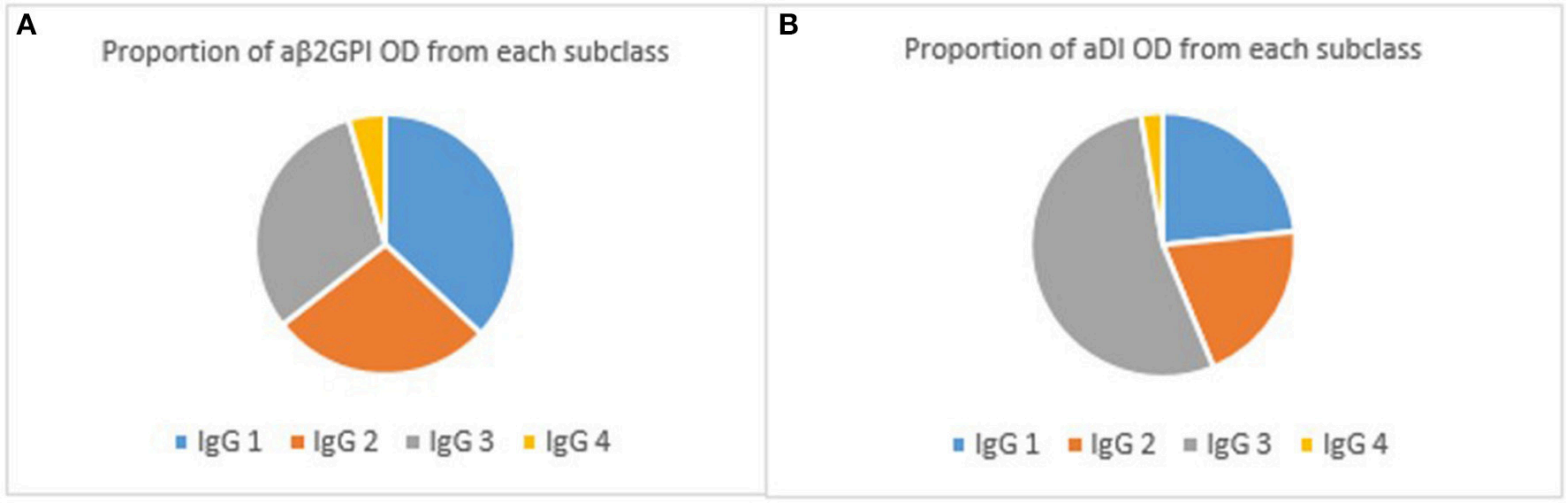
**(A)** shows the proportion of aβ2GPI OD associated with each subclass. As can be seen, IgG1 predominates in the aβ2GPI assay whilst IgG2 and IgG3 are similar. **(B)** shows the same proportions for aDI subclasses. It is clear here the predominant subclass is IgG3.

IgG4 was the lowest detected subclass in both aβ2GPI and aDI (*p* < 0.05 across all comparisons). In aβ2GPI assays, the IgG1 subclass was the most predominant, followed by IgG2 and IgG3, with no statistically significant difference between these three subclasses (Table 2, Figure 1A, *p* > 0.05 across all comparisons). In aDI assays however, the percentage of IgG3 was significantly higher than both IgG1 and IgG2 (Table 2, Figure 1B), demonstrating that in our patients, aβ2GPI and aDI can be of a different subclass. The average percentage of cumulative binding for all patients for each IgG subclass is summarized as a pie distribution chart in Figure 2 (2A, aβ2GPI and 2B, aDI). Raw OD values can be seen in the Figure S1.

Analysing the patients individually, 12/18 patients positive for aDI had the majority of their antibody binding specific to the IgG3 subtype. In contrast, only 4/18 had the highest for IgG1. In the aDI assay, over 50% of the cumulative total OD was seen in the IgG3 subtype for 9/18 patients, and 4 of these patients showed over 75% of their cumulative antibody binding to be of the IgG3 subclass. Comparatively, only 1/18 showed >50% of the cumulative binding for IgG1 subclass (51%) and no patients showed >50% of cumulative antibody binding for either IgG2 or IgG4. In contrast, when analyzing the aβ2GPI subclass pattern, only 3/19 patients had >50% antibody binding for IgG3 whilst 7/19 has >50% cumulative antibody binding for IgG1, and one patient showed >50% cumulative antibody binding in IgG2.

**Figure 3:**
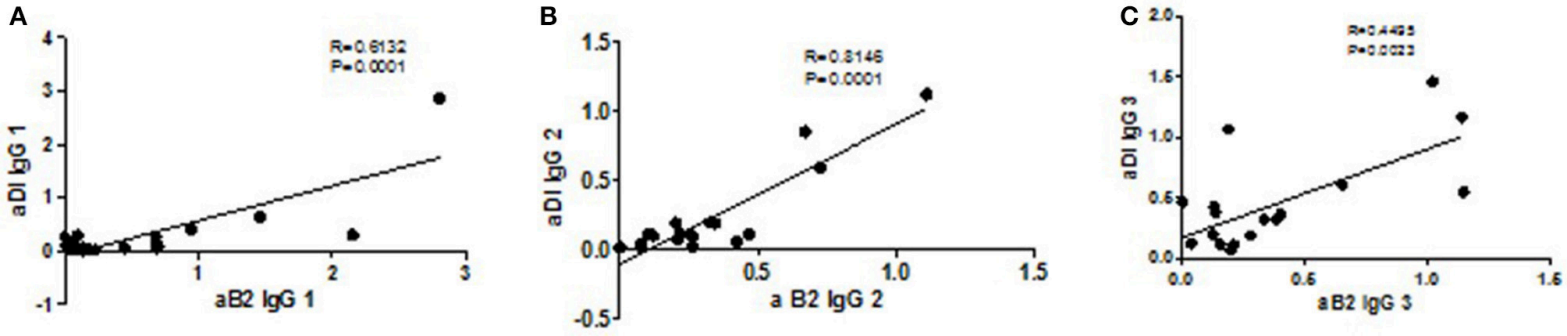
Correlations of subclasses between antigens. The strongest correlations are seen for IgG1 (**A**, *p* = 0.0001, *r* = 0.61), IgG2 (**B**, *p* = 0.0001, *r* = 0.81) and IgG4 (*p* = 0.0001, *r* = 0.85; data not shown). The weakest correlation is seen with the IgG3 subclass (**C**, *p* = 0.02, *r* = 0.45) reflecting the difference between the antigens in the IgG3 distribution.

Correlation analysis of each IgG subclass for the two antigens revealed strong correlations between aβ2GPI and aDI of the same subclass for: IgG1 (*r* = 0.61, Figure 3A), IgG2 (*r* = 0.81, Figure 3B) and IgG4 (*r* = 0.85, data not shown) The difference is highlighted in IgG3 where the correlation is much lower (*r* = 0.45, Figure 3C), reflecting the increased level of IgG3 aDI antibodies compared to the IgG3 aβ2GPI antibodies.

When stratified by clinical phenotype (thrombosis vs. pregnancy morbidity), the results were very similar across all IgG subclasses, with no clear association between a single subclass and clinical history (data not shown). However, this may be confounded by low patient numbers.

### Total levels of each IgG subclass were similar in patients with APS than controls

**Figure 4:**
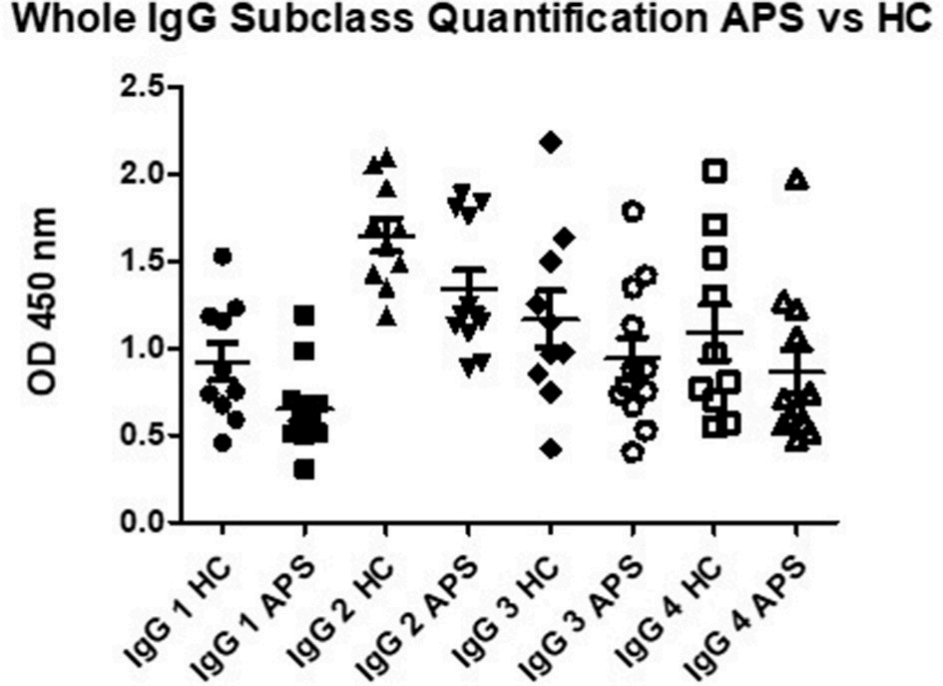
Total capture IgG ELISA results demonstrate that there are no significant differences in OD for IgG 2, IgG 3 and IgG 4 subclass in comparison of APS patients to healthy controls (HC). Subclass IgG 1 shows significantly lower levels in APS, however, removal of the outlier in the HC group shows no significant difference between APS and HC.

To exclude the possibility that the high IgG3 aDI levels in patients with APS were simply a reflection of over-production of total IgG3, we measured total levels of each subclass in 10 healthy controls and 12 patients with APS. As can be seen in Figure 4, there are no significant differences between the two groups for total IgG 2, IgG 3 and IgG 4, thus underlining that the high IgG3 aDI levels we detected in patients are indeed antigen-specific. IgG 1 was significantly different between APS and HC, with lower levels in HC (*p* < 0.045) however, removal of the outlier in the HC group (1.53) loses the significance.

## Conclusions

Our data suggests that antibodies to DI are of a different subclass to antibodies against whole β2GPI. In our cohort, antibodies to DI are predominantly IgG3, whilst antibodies to β2GPI are more prominently IgG1.

Our results conflict with previous data, which suggested an IgG2 class restriction for aβ2GPI antibodies. These studies however, were 20 years ago and our ability to characterize subclasses of IgG has improved, as has the methodology for detecting antibodies to both DI and β2GPI. The production of subclass specific monoclonal antibodies has improved and aβ2GPI antibody assays have been developed and refined in that time, although no consensus on the source of antigen exists. Another possibility for the discrepancy between our results and others', is that by selecting for patients who are both aβ2GPI and aDI positive, we have isolated a patient population with less overall IgG2 aβ2GPI and an overall different serological profile. We knowingly selected patients with positivity to both β2GPI and DI, allowing us to directly compare the subclasses detected in the two assays. The dominance of IgG1 and IgG3 subclasses is interesting as these are heavily involved in complement activation, particularly IgG3, and may offer an insight to the mechanisms of pathogenesis in APS. Interestingly, a difference in subclass between these antibodies may imply several mechanisms are active in APS patients. The IgG subclasses differ in their affinity to receptors, their half lives and their potential activity *in vivo*.

It was expected that the aβ2GPI and aDI subclasses would be similar, as β2GPI contains the aDI epitope, however, the results are quite disparate. This would suggest that the aβ2GPI assay does not necessarily detect all circulating aDI antibodies, and indicates a clinical utility for the aDI assay in patients. Indeed, recommendations for development, standardization and application of aDI assays in APS have existed for some years now, first highlighted at the 14th International Congress for Antiphospholipid Antibodies (35). Of note, we reported good qualitative and quantitative agreement between our IgG aDI ELISA and the USDA approved chemiluminescent assay for aDI, developed by Inova Diagnostics (36). Despite this, it is possible that the structure or presentation of the protein on the plate may have influenced the results presented. Ideally this would be repeated in the fluid phase to confirm binding to subclasses.

To guard against bias from abnormally high or low IgG subclass concentrations, patient antibodies were tested along with healthy controls the results of which showed similar proportions of IgG subclass antibodies between patients and controls. It could be argued that the use of another irrelevant antigen would aid in proving that the effect seen is not just a skewing of antibody production, however, we felt that (a) given the aDI and aβ2GPI subclasses differed, and (b) similar quantification was seen in the total IgG subclass ELISA, that this was not required.

To conclude, our findings suggest that IgG aDI are of a different subclass to aβ2GPI. While unexpected, our results may demonstrate IgG subclass switching of aβ2GPI to aDI, or vice versa, and thus epitope restriction or spreading respectively. Importantly, our study highlights the potential for aDI testing to stratify patients. Further studies are required to validate these findings in larger patient cohorts. The ability of IgG1 aβ2GPI vs. IgG3 aDI to exert pathogenic biological effects also warrants investigation, as autoantibody skewness to specific IgG subclasses may indicate an association of these antigen-specific antibodies with clinical outcomes.

## Ethics statement

This study was carried out in accordance with the recommendations of London Hampstead Research Ethics Committee Ref No 12/LO/0373 with written informed consent from all subjects. All subjects gave written informed consent in accordance with the Declaration of Helsinki. The protocol was approved by the London Hampstead Research Ethics Committee Ref No 12/LO/0373.

## Author contributions

The project was conceived by TM and CP. BA-E supplied patients, CW, DI, CP, IG, AR, and VR gave intellectual advice. CW also provided data for the review process.

### Conflict of interest statement

TM, CP, IG, RA are inventors on a patent for Domain I in the US. The remaining authors declare that the research was conducted in the absence of any commercial or financial relationships that could be construed as a potential conflict of interest.

## Supplementary material

Figure S1 Panel **(A)** shows aβ2GPI levels as raw OD for each of the subclass specific secondary antibodies. **(B)** mirrors the aβ2GPI assay in an aDI assay.
